# The culture of official statistics. Symbolic domination and “bourgeois” assimilation in quantitative measurements of immigrant integration in Germany

**DOI:** 10.1007/s11186-022-09503-2

**Published:** 2022-10-21

**Authors:** Martin Petzke

**Affiliations:** Faculty of Sociology, Bielefeld University, Universitätsstr. 25, 33615 Bielefeld, Germany

**Keywords:** Culture, Pierre Bourdieu, Quantification, Social Inequality, State, Symbolic domination

## Abstract

While cultural sociology has recently made a comeback in research on social inequality both in the context of poverty studies and studies of immigrant integration, it has rarely investigated how particular constructions of the problem of socioeconomic mobility are themselves culturally situated. The article addresses this neglect by investigating the problematization of disadvantaged lives within the relational framework of Bourdieu’s cultural theory of the state. Here, the state exercises symbolic violence by transforming one arbitrary cultural standpoint in social space into a universal standard, or a taken-for-granted “doxa,” against which other cultural positions can only come off as deficient. The article extends this perspective by addressing the role of official statistics in this process. Taking Germany’s official monitoring of the socioeconomic integration of immigrants as its case and drawing from document analysis, interviews, ethnographic observation, and data from the German General Social Survey, the article shows how such statistical instruments of the welfare state in fact tacitly universalize a model of the good life particular to civil servants, the very constructors of the monitors, as a benchmark for immigrant integration.

Exponents of cultural sociology have more recently made a conscious effort to extend cultural analysis to a broader gamut of social phenomena (Binder et al., 2008) and to expand its conceptual repertoire beyond a Parsonian preoccupation with norms and values (Lamont & Small, 2008). Such maneuvers have once again opened up provocative cultural perspectives on a central concern of sociology: social inequality. Adding depth and nuance to the various iterations of the culture-of-poverty hypothesis, which deems the reproduction of poverty a result of cultural adaptations to such conditions (Lewis, 1966; Massey & Denton, 1993; Ogbu, 1978; Wilson, 1987), cultural sociologists have shed new light on how the poor navigate and make sense of their lives (see, e.g., the contributions in Harding et al., 2010).

This trend has been flanked by a renaissance of cultural perspectives in the sociology of immigration, a field similarly driven by concerns with social inequality. While culture figured prominently in Gordon’s (1964) seminal work on immigrant assimilation, the latter has largely come to be operationalized by indicators of socioeconomic mobility once quantitative perspectives had entered the field in the 1970s (Alba & Nee, 2003, p. 28; Skrentny, 2008, p. 67). Cultural perspectives have made a forceful comeback, however, in more recent theories of assimilation.

Alba & Nee (2003), for instance, employ an “institutional framework” that gives prominence to “mental models shaped by cultural beliefs” (ibid., p. 37), to “human-cultural capital” (ibid., p. 48), and to values and broader institutional changes (ibid., p. 57) in analyzing assimilation. Like Zolberg & Woon (1999), the authors furthermore stress the significance of boundaries – i.e., their blurring, crossing, and shifting – in processes of immigrant assimilation and absorption, adopting a concept that has similarly informed recent work on morally imbued distinctions of race, class, and immigrants (Lamont, 2000).

Cultural arguments have also figured prominently in the theory of segmented assimilation (Portes & Zhou, 1993). It posits that some immigrants may culturally assimilate not to the mainstream but to the urban underclass and its “oppositional culture” (a term coined by Ogbu, 1978), thus forgoing social advancement, while still others may achieve upward mobility precisely by not assimilating but by drawing on the cultural resources and orientations of their ethnic enclaves.

Lee & Zhou (2015) have moreover explicitly latched on to resurgent cultural analyses of social inequality in investigating the factors behind Asian American achievement. They show how a “hyper-selection” of highly educated Asian immigrants imports and consolidates a “success frame” and attendant institutions from which co-ethnics with different class backgrounds also benefit.

Yet, as cultural sociology has reclaimed a prominent position in exploring and explaining social inequality, it is still marred by a considerable neglect. In calling for “bolder, deeper, and broader” cultural analyses of socioeconomic mobility in relation to race, ethnicity, and immigration, Skrentny (2008, pp. 68–69) notes that investigations into the role of minority cultures have rarely been couched in a relational framework with an equal focus on the culture of the majority (but see Jiménez 2017; Lamont, 2000). Indeed, the middle-class or whites have largely constituted a blind spot in what Streib et al. (2016) have more recently criticized as a “one-sided research agenda.” It is informed by a taken-for-granted and uncontested binary “whereby segments of the poor, racial minorities, and immigrants are positioned as having a deviant, morally suspect culture that undermines their potential upward mobility whereas white middle- and upper-class Americans are positioned as having a normal, morally upstanding culture that secures their class position” (ibid., p. 248). In many ways, the culture of the latter is the “unmarked” side of the distinction that remains unproblematic and thus underexplored (Brekhus, 1998).

Indeed, a more emphatic focus on the particularity of the dominant cultural standpoint could cast further doubt on an improbable assumption that recent cultural analyses of social inequality have only begun to dismantle: “[I]s it likely, or even possible, that all groups on average want all the same things out of life, in the same degree, and balance competing demands in the same ways? Does everyone see the same meaning in life?” (Skrentny, 2008, p. 69; see, e.g., Sanchez-Jankowski, 2008; Vaisey, 2010; Young, 2010).

What is more, such directions in research would stand to benefit from a thoroughly relational perspective that examines the construction of *the problem of inequality* within a comprehensive field or ecology of class positions. To be sure, some have turned the analysis back on the welfare discourse of political elites (Guetzkow, 2010; Somers & Block, 2005; Steensland, 2006). Yet, as such work highlights frames of deservingness and undeservingness, it continues to fall short of fully objectifying the cultural standpoint from which such frames are constructed. It still leaves open the question: how is the way policy elites make sense of *their* life implicated in the way *they* make sense of the lives of the poor?

In her historical study on *Poverty Knowledge*, O’Connor (2001, p. 23) has touched on such relational perspectives in highlighting “that poverty knowledge has been filtered, not just through the experiences and cultural biases of the privileged, but through the social position of ‘the professors’ in relation to the ‘poor’” (see also Gans, 1991, p. 299). This points towards the need for a systematic sociological exploration of such filtering processes. It calls for a cultural grounding not of social inequality itself but *of the epistemology* of observing and problematizing inequality.

The article thus seeks to recast the light on the cultural premises through which certain lifestyles are deemed problematic in the first place. As already indicated by the first steps undertaken by the scholars mentioned above, such a focus inevitably falls on two major institutions involved in constructing the problem of social inequality: the welfare state and the social-scientific community. Both have more recently been the subject of scrutiny regarding implicit normative commitments inherent in purportedly neutral perspectives on socioeconomic (im-)mobility. For the welfare state, Steensland (2010) has made a broader case to consider the connection between morality and social policymaking. For the social sciences, Abbott (2016) has highlighted tacitly bourgeois conceptions of standard life trajectories in quantitative research on inequality.

Taking their insights as a cue, this article focuses its inquiry on a case in which official perspectives of the state as well as social-scientific methods of inequality research intersect. In Germany, administrative bureaucracies on the municipal, state, and federal level have resorted to social-scientific expertise and quantitative research methods in order to monitor the social advancement of the immigrant population. In so-called “integration monitoring systems,” compilations of socioeconomic indicators derived from official statistics are used to compare the population segments of migrants and non-migrants in order to effectively govern the institutional incorporation of first- to third-generation immigrants. The statistical parameters include level of education, unemployment rates, income, health, household size, area of living space per household member, rate of real estate ownership, fertility rate, civic engagement, and political involvement. In line with much of social inequality research and the sociology of immigrant integration, policies oriented by these statistics seek to close the gap between both population segments, or, in other words, to eliminate the systematic effect of a migration history on these socioeconomic indicators. An analysis interested in the cultural grounding of the epistemological apparatus of observing social inequality and socioeconomic mobility would have to attend precisely to such a statistical monitoring instrument.

And indeed, I argue that the indicators of the monitoring instrument tacitly impose a class-culturally particularistic ideal of a typically “bourgeois” life. They do so first and foremost through their choice of variables. Inevitably, the fact that a certain dimension is measured already bestows significance on the dimension itself. The specific configuration of indicators inadvertently produces a “grid” of a particular biographical standard. Thus, normative implications emanate not only from measuring and publicizing statistical “norms” as captured by the means or medians of various parameters; they are also, and perhaps more fundamentally so, conveyed by the very fact that some aspects are deemed “worthy” of measuring in the first place. Alternative conceptions of the “good life” that would lead to entirely different measurements are thus eclipsed or manifest themselves only as deviations from the statistical averages of the selected indicators. Epistemologically, then, the statistical perspectives of the monitors exhibit not just biases but *erasures* as they standardize the matrix through which images of immigrants are filtered.

The argument builds on the relational perspectives of Pierre Bourdieu, specifically his more recently published theory of the state, which he developed in a lecture course at the Collège de France between 1989 and 1992 (Bourdieu, 2014). As I will demonstrate, integration statistics espouse cultural standards that have a specific location in social space. Among all occupational groups, they are most pronouncedly exhibited by civil servants with executive duties (*Beamte im höheren Dienst*), i.e., the occupational group that in Germany includes department heads of administrative bureaucracies and university professors. In many regards, then, the very people who construct indicators of integration are unwittingly universalizing their personal ideas about the good life as a standard for successful integration. The cultural heterogeneity even of the national mainstream is thus obscured, and the presence of alternative or subcultural ideals and aspirations is removed from the statistical picture.

It is precisely this symbolic dimension of statehood that Bourdieu brings to the fore in his theory of the state. For Bourdieu, the state holds not just the monopoly of legitimate physical violence but also of legitimate symbolic violence: It monopolizes the means to transform one particular standpoint among many into a *doxa*: a reality prereflexively recognized as natural, self-evident, and without alternative. This doxa, however, is merely masking an orthodoxy that could in principle be challenged.

I argue that official statistics on integration can be understood as an example of how the state exercises its monopoly of legitimate symbolic violence and how it establishes the doxa of a “normal” biography. These statistics construct as well as reflect taken-for-granted schemes of perception that determine which questions can and cannot be asked about social inequality in the first place and what can actually be *seen* when problematizing the life of others. In so doing, they at the same time obscure the particularity of their own perspective.

The article thus adds to a growing literature on the state that has taken a cue from Bourdieu in examining the cultural backdrop of state action (e.g., Loveman, 2005; Morgan & Orloff, 2017; Norton, 2014; Steinmetz, 2007). It extends such work in drawing specific attention to the process in which state actors implicitly universalize their particular habitus as a model of the good life in the context of observing and addressing socioeconomic inequality and mobility from the perspective of the welfare state.

The article will proceed as follows. In a first section, I introduce Bourdieu’s cultural theory of the state and articulate it with a literature on the sociology of statistics and quantification. I then briefly discuss the administrative monitoring of immigrant integration in Germany. After a section on data and methods, I highlight the process through which civil servants on the municipal, state, and federal level select and compile indicators of integration. I proceed with an ideal-typical depiction of German bourgeois culture and briefly highlight how civil servants were not only among its most central carriers but also the pioneers of social surveys in the context of the worker question. Crucially, such surveys were part of a broader agenda that *explicitly* understood the institutional incorporation of workers as a project of *cultural* assimilation to the bourgeois lifestyle. I argue that this historical legacy continues to reverberate in contemporary official statistics, though now implicitly so, not least because their production and maintenance is still situated at the very same location in “social space” (Bourdieu, 1984), occupied by the most senior ranks of civil servants. I proceed to highlight the implicitly bourgeois presuppositions in the integration monitoring, composed of such social statistics, and corroborate my argument with data from the General Social Survey of Germany, or the ALLBUS. Using quantitative tables, I show that *even among German nationals alone* the different occupational groups exhibit substantial differences on these indicators, with civil servants – or the *Beamtenbürgertum* as a particular segment of the educated bourgeoisie – *most closely* conforming to the model of life standardized by their own statistics. I end with a discussion on the broader implications of these findings.

## Bourdieu’s theory of the state

Bourdieu’s theory of the state can be encapsulated in a definition that extends Weber’s classic definition of the state as the monopoly of legitimate physical violence. For Bourdieu (2014) the state also needs to be understood as the monopoly of *legitimate symbolic violence*.

Despite the central position of struggle, antagonism, and competition in Bourdieu’s social ontology, his theoretical edifice is to no small extent a theory of symbolic order. His work is as indebted to Durkheim as it is to Weber, and in this regard the idea of moral *and* logical conformity in the integration of society has been central to Bourdieu’s theory. Individual habitūs in social space incorporate shared “principles of vision and division” and a practical understanding of the differential legitimacy of particular positions and lifestyles. Such perceptions are differently inflected and weighted according to each agent’s position within social space (Bourdieu, 1984, p. 468). The unique coloring each habitus lends to such classifications translate into feelings of entitlement, confidence, and repulsion, or, conversely, respect, resentment, self-consciousness, and shame. This is the basis of symbolic violence: The dominated participate in and assent to their own domination as they themselves perceive the world through classificatory schemes which, being the product of such domination, are themselves constitutive of it (Bourdieu, 2001).

However, this conception of the social space leaves open the question of how, in fact, moral and logical integration are guaranteed. After all, Bourdieu disassociates himself from structuralism in what he calls “genetic structuralism” (Bourdieu, 1990, p. 14). Classificatory schemes are historical products and they are amenable to change through “symbolic revolutions” (Bourdieu, 2017). How, then, is such a comprehensive symbolic order at least temporarily established?

It is here where the state comes in. In fact, as Bourdieu (2014, p. 223) acknowledges retrospectively, when he had been writing of the social space as the “space of spaces” or “field of fields,” all along he had been in fact referring to the “national social space that is constructed at the same time as the state is constructed, that the state constructs as it constructs itself.” For Bourdieu, the key function of the state goes beyond territorial and economic integration; it lies as much in the unification of the symbolic order, or of the “market in symbolic goods.” (Bourdieu, 2014, p. 225). This unification, however, simultaneously amounts to the universalization of a particular standpoint. Such a monopolization of the universal, or the “double face” of domination and integration, forms the central characteristic of Bourdieu’s (2014, pp. 222–223) conception of the state. Processes of integration and unification are intimately tied to processes of subjugation and dispossession as “non-official cultures [are] made into more or less accomplished forms of the dominant definition of culture” (ibid., p. 223).

Bourdieu touched upon this crucial operation of a unification of the symbolic market in earlier studies, for instance in his 1970 study on social reproduction (Bourdieu & Passeron, [1970]1990), his work on *Language and Symbolic Power* (Bourdieu, 1991), and several articles on the matrimonial market of Béarn, the earliest of which dates back to 1962 (Bourdieu, 2008). In each of them, he shows how one cultural arbitrary is imposed as the legitimate standard, effectively devaluing all others, be they French dialects other than the Parisian one in the unification of the linguistic market or rural life as opposed to urban life in the unification of the market of lifestyles. Only later in his lecture course does Bourdieu (2014) finally identify the state as the entity driving such processes of monopolization and symbolic domination; it is the fundamental element of his sociology of modern society which until then had been left largely unspecified.^1^

For the state, official statistics, i.e., “statistics that governments produce, finance, or routinely inscribe into their decisions” (Starr, 1987, p. 8), are obviously a crucial element in imposing a symbolic order on the social world. It is here where Bourdieusian perspectives can be fruitfully articulated with a literature on the sociology of quantification that takes numerical practices and statistics as an object of investigation in its own right (for a recent overview see Mennicken & Espeland, 2019).

As early proponents of such an agenda have pointed out, official statistics have the power to “change images, perceptions, and aspirations” simply “[b]y the questions asked (and not asked), categories employed, statistical methods used, and tabulations published” (Alonso & Starr, 1987, p. 1). Moreover, “political judgments are implicit in the choice of what to measure, how to measure it, how often to measure it, and how to present and interpret the results” (ibid., p. 3).

It is precisely such implicit judgments which lie at the heart of this article. Such judgments, I argue, to no small degree derive from state officials’ class-based conceptions of the good life. It is in this vein that Bourdieu advises us to “relate the categories or classification systems used to the users and originators of these classifications and to the social conditions of their production” (Bourdieu, 2004, p. 90). Work in the sociology of quantification has already fruitfully highlighted the constitutive role of class background in the invention of particular methods of statistical analysis (MacKenzie, 1981). Bourdieusian interests in the social conditions of social knowledge production have also served as an inspiration for Alain Desrosières’s (1998) seminal turn to the history and sociology of quantification. His work, specifically, highlights the “conventions” and historical contexts that underlie the creation of statistical objects of national statistics such as unemployment rates. Here, social interests have figured prominently in the discussion of the genesis of socioprofessional categories (Desrosières, 1998, pp. 267–273). The latter perspectives connect to previous studies which, in experimental reenactments of coding situations, have shown that an individual’s position in social space has a substantial impact on how she constructs occupational classifications and which occupation she considers representative (Boltanski & Thévenot, 1983; Desrosières & Thévenot, 1988).

The present paper builds on such work but brings it into a deeper conversation with Bourdieu’s theory of the state. I show that official statistics are a central means through which state officials universalize a particular view of the social world from a specific location in social space. Specifically, I argue that through the construction of integration indicators, bureaucrats of the welfare state and social scientists who advise them implicitly universalize a perspective rooted in the habitus of a particular segment of the educated bourgeoisie, i.e., civil servants with executive duties, which, in Germany, include heads of administrative departments and university professors. Official statistics and social-scientific indicators compiled by administrators for the purpose of identifying and addressing inequalities contribute to a specific doxa of what constitutes a standard biography. It is a conception of the good life which is in fact particular to civil servants. Effectively, in determining what is “worth” measuring and what is not, statistics form a substantial yet undertheorized cog in the unification of a symbolic market of lifestyles.^2^ In implicitly imposing the relevant measures of a standard biography, they eclipse and obfuscate alternative cultural perceptions of the good life that would call for entirely different indicators. In so doing, statistics aid in transforming “orthodoxy” into “doxa,” the key operation in the monopolization of legitimate symbolic violence (Bourdieu, 2014, pp. 173–174). Quantification and indicators in fact form a powerful mechanism of such an obfuscation of alternative conceptions of worth, given that through their “aura of objectivity” (Merry, 2011; Porter, 1995) the very act of obfuscation is itself obfuscated.

## Case and methods

The study is part of a larger investigation into how quantitative indicators affect official perspectives on immigrant integration in Germany. Over the last two decades, municipalities, states, and the federal government in Germany have increasingly installed statistical instruments for the purpose of monitoring immigrant integration. This development is related to a shift in immigration policy ushered in by the Social Democrat/Green Party coalition that came to power in 1998. Germany was now officially considering itself a country of immigration, putting an end to the guest-worker paradigm that had prevailed before. With the recognition that immigrants and their descendants were here to stay, the question of their integration now became a central part of political discourse.

The initial impulse to employ statistical indicators in monitoring and addressing integration problems came from the municipal level, with some communities pioneering such measures in the early 2000s. State administrations soon followed suit and established a joint working group that has maintained a state-level integration monitoring since 2008. Some states have also developed their very own and often more extensive integration monitorings. On the federal level, the office of the Commissioner for Migration, Refugees, and Integration published two pilot studies on integration indicators in 2009 and 2011. Some of these indicators had already appeared in the Commissioner’s regular reporting on the situation of “foreigners” since 1978. These reports, which meanwhile have also shifted to a more comprehensive focus on first- to third-generation migrants, are now featuring many of the new indicators tested in the pilot studies. In 2020, the Commissioner moreover launched its first official integration monitoring report as a separate publication (see Commissioner for Migration, Refugees, and Integration, 2009, 2011, 2020).

All integration monitorings in Germany generally follow the same basic pattern. They draw upon official statistics and registers of the various governmental departments and the general social surveys in Germany such as the microcensus and the Socioeconomic Panel (SOEP) for indicators, which include levels of education, unemployment rates, income, health, household size, area of living space, rate of real estate ownership, fertility rate, civic and political engagement, and others (see Table1).

**Table 1:** Typical integration indicators in municipal, state, and federal integration monitors in Germany^a^

*Education* Pre-K enrollment Enrollment in secondary schooling by type Enrollment in vocational training University enrollment Highest educational degree Completed vocational training Language skills	*Employment* Employment rate Self-employment rate Enrollment in further occupational training Atypical and casual employment Long-term unemployment Net income Poverty risk
*Living conditions* Average living space per household member Percentage living in self-owned real estate	*Legal Status* Naturalization rate among the eligible population Type of residence permit
*Health* Obesity rate Basic vaccination among children Use of preventive health care measures for children Use of preventive health care measures for adults Frequency of exercise	*Family* Family structure Household size Percentage living in households of 5 or more Number of births per female Intercultural marriages Culturally mixed households
*Social Integration* Political engagement in parties and local initiatives Membership in voluntary associations Engagement in volunteer work Engagement in executive positions in voluntary associations Intercultural contacts in private settings	*Crime* Crime rate Violent crime rate

^a^ Groupings and headers may vary among the various integration monitors

Such indicators generally compare averages between the native population and the immigrant population, except where such comparisons are not applicable (as in rates of naturalization). For such comparisons, the monitors employ a distinction between persons with and persons without a “migration background,” the first including all people not born as a German or with at least one parent not born as a German. Negative discrepancies between both segments are interpreted as an integration deficit.

**Fig. 1:**
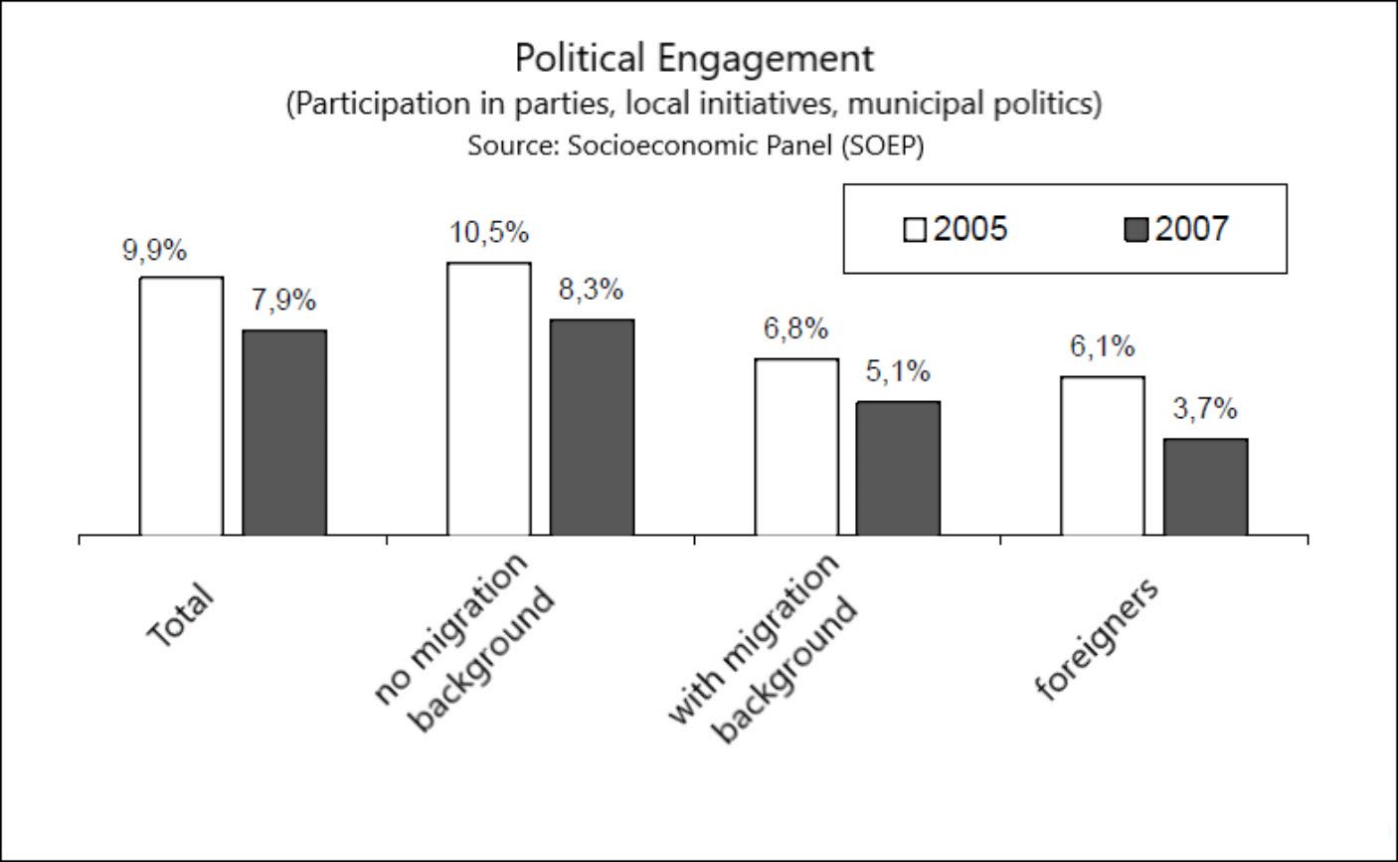
Example of an integration indicator; own adaptation from Commissioner for Migration, Refugees, and Integration (2009, p. 95)

Although these monitorings generally feature information on the distribution of immigrants according to legal status of residency, occupational qualifications, and country of origin that reflect current immigration policies, the integration indicators themselves very rarely make such distinctions.^3^ At stake is simply the “measurement of the current state of integration through an equalization of the chances or living conditions, respectively, in various sectors (education, work, housing),” a formulation set down in the National Integration Plan, the Federal Government’s (2007, p. 121; my translation) authoritative statement of its integration policy. Essentially, then, the integration monitorings employ the basic quantitative methods of the sociology of social mobility and of immigrant incorporation, comparing the native mainstream with first- to third-generation migrants on central indicators of socioeconomic and civic life (for a recent overview see Drouhot & Nee, 2019; for a critique see Wimmer & Glick Schiller, 2003). Fig.1 shows an example of a typical integration indicator for political engagement.

In exploring how such quantitative perspectives shape the construction of the problem of immigrant integration, I investigated several units embedded within the case of Germany (see Gerring 2004; Yin, 2014 on embedded single-case designs). On the municipal level, I focused on a middle-sized city which was among the first to design and publish a statistical integration monitoring. It shall here go by the pseudonym Stadthausen. On the state-level, I looked at a territorial state in the western part of Germany, here referred to as Westlanden, which has developed its very own monitor that by far extends the core indicators of the joint-working group among states. It also sponsors a program that promotes the implementation of integration monitors in all communities within the state, which provided further data from the municipal level. On the federal level, the focus was on the office of the Commissioner for Migration, Refugees, and Integration.

The study draws on a range of methods. I employed a document analysis of integration reports and related publications on all governmental levels. The corpus contained over 70 documents from five municipalities, from one state and the state-level working group, as well as from the office of the Commissioner for Migration, Refugees, and Integration.

Document analyses were complemented by 25 semi-structured in-depth expert interviews and 14 informal expert interviews with administrators, politicians, and project coordinators on the federal, state, and municipal level, conducted between June and December of 2017. Semi-structured interviews lasted between one and two hours. The interviews aimed to assess how quantitative indicators of integration are chosen and compiled and how they are involved in governing integration.

Third, I conducted ethnographic observation of official integration conferences, administrative working groups and executive committees for integration, intermunicipal network meetings between integration officers and actors in the field of integration work, and sessions of political committees for integration. These observations, which took place between October 2017 and June 2018, served to shed additional light on how quantitative indicators are implicated in the ways in which state officials construe the problem of immigrant integration in Germany.

Documents, interview transcripts, and field notes were coded following the central tenets of grounded theory (Charmaz, 2006) and using atlas.ti. I used an open and iterative approach to identifying main themes and particular ontologies of the social. Comparing data and shifting back and forth between data and analysis, I discovered specific valorizations of lifestyles in the particular selection of indicators of immigrant integration, which I related to historical research on German bourgeois culture (see sections "Bourgeois culture in Germany" and “The bourgeois undercurrent of monitoring integration” below). Abductively informed by such research, I condensed such valorizations into more general bourgeois value-complexes which encompass most of the indicators in the monitoring reports. All evidence from the document analysis, interviews, and ethnographic observation has been translated from German into English.

Finally, in order to show that indicators in fact universalize a conception of the good life particular to civil servants with executive duties as a segment of social space, I use data on occupational groups among German nationals from the pooled data set of the ALLBUS (1980–2014), the German General Social Survey. This data set provides large enough Ns to allow for the segmentation into occupational meta-categories. I construct simple tables instead of conducting regressions with the usual controls. I do this for a fundamental reason. Following Bourdieu, the analyses are concerned with the structure of social space and the various forms of habitus it brings forth. Here, combinations of education, income, geographical location, sex, ethnic origin, and even age are seen as crucial determinants (Bourdieu, 1984, pp. 99–106). Occupational position is thus used as a proxy for these determinants and not understood as an independent variable. In this, I follow the typical conventions of Bourdieu’s own analyses (see, for instance, the statistical appendix in Bourdieu 1984, pp. 525–545). As in the work of Bourdieu, the tables will show that, in comparison, particular occupational groups are overall *more likely* to exhibit a penchant for specific forms of practice, without denying significant internal variation within the group. The analysis shows that what today’s civil servants understand as a well-integrated life according to their own measures of integration is in fact the life to which they themselves conform most closely, with much deviation in other segments of the social space even when looking at German nationals alone.

## Selecting the indicators

On all governmental levels, the procedure of compiling integration indicators follows a very similar pattern. In Stadthausen, which established one of the first integration departments in Germany after the paradigm shift in immigration policy, the first head of the integration office chose to assemble the various heads of other departments pertinent to integration policy (i.e., social affairs, housing, statistics, etc.) in an “executive committee” for integration. As the current head of the integration department (who was also a founding member of the executive committee in her position as the head of a unit within the department) remembered, the indicators were (and still are) born out of discussions within this group:

We in the executive committee, which includes [the head of the statistical office] and the other department heads, have always discussed the indicators together…We devised the basic indicators in close coordination [with the statistical office], and, naturally, over time we often had thoughts such as ‘oh, that could be interesting, can that be measured,’ and then [the head of the statistical office] went out and thought about it.

On the federal level, the selection of indicators likewise occurs in close coordination and conversation with civil servants from other ministries and departments who maintain their own indicators. Here, too, the federal statistical office is intricately involved in the process. As the responsible civil servant put it: “The question is: what are meaningful indicators from our perspective?” Indicators from other sources are, as she phrased it, “read against the grain.”

In Westlanden, the head of the unit (*Referat*) responsible for designing the monitoring painted a similar picture of concerted deliberation: “We wanted to do a bit more [than the joint-working group of German states]. And since we had the human resources, we sat together, really in the sense of a ‘brainstorming,’ thought about different things, looked at different data sources whether there was something relevant to be found.”

Often the expertise of another group of higher civil servants is enlisted in compiling integration indicators: university professors. Stadthausen sought the advice of a local institute affiliated with the European Forum of Migration Studies at the University of Bamberg, then led by the sociologist Friedrich Heckmann, an expert on migration and integration who since the 1990s has served as a government consultant on immigration affairs. Both Westlanden and Stadthausen employ Heckmann’s (1997) theoretical dimensions of integration (structural, cultural, social, identificatory) as a broader skeleton in presenting the various indicators in the monitoring reports. Heckmann also evaluated many of Westlanden’s integration programs. On the federal level, sociologists of the Berlin Social Science Center (WZB) were involved in the publication of the two pilot studies on integration indicators and provided multivariate analyses (Commissioner for Migration, Refugees, and Integration, 2009, 2011).

All civil servants interviewed espoused a very clear objective behind the integration monitoring, or rather the policies it seeks to inform. For them, the aim was an “equalization of life chances,” a formula mentioned not only in virtually all monitoring reports but also, as seen, in the Federal Government’s National Integration Plan. As the officer in charge of Westlanden’s monitoring put it, “assimilation is not the goal, we want equal chances of participation.” A member of Stadthausen’s executive committee phrased the key question behind the monitoring thus: “Are they [i.e., persons with a migration background] getting to where the host society is”?

Yet, despite such clear-cut perspectives regarding the purpose of the integration monitoring, the actual process of settling on a specific set of indicators seems to be a rather intuitive one. For the responsible civil servant on the federal level, the preference was above all for “structural” indicators since these policy areas usually lie within federal jurisdiction, and for indicators that permit the construction of time series. On the state level, too, the respective administrator spoke of a rather inductive and, as she put it, “pragmatic” process in selecting indicators: “One makes do with what one has. If we did it differently, asking ‘what do we need,’ we would have so many gaps that the whole affair would be a rather unsatisfying process.” Indeed, the framework adopted from Heckmann seems to provide only a loose heuristic since indicators are sometimes reassigned to other dimensions over the publication intervals without administrators being able to recall the precise reason.

The selection of indicators is thus hardly reflected as a value-laden or culturally impregnated procedure. Discussions among civil servants are generally preoccupied by far more minute aspects of an issue. In response to my question regarding past controversies, one civil servant from Stadthausen merely recalled a debate on whether the indicator on real-estate ownership should also include real-estate not lived in by the owner him- or herself. The broad strokes of the compilation, in contrast, seem to come off as rather uncontroversial and self-evident to those involved in designing the monitoring.

To wit, when I asked why civic engagement in voluntary associations is included as an indicator of integration, the head of Stadthausen’s department said: “Civic engagement has something to do with contributing to society, feeling responsible, that’s why it was always an interesting field”; similarly, the head of Westlanden’s integration department pointed to a “civic sense of responsibility,” using a German term for “civic” that carries a little more gravitas (“staatsbürgerlich”). This stood in stark contrast to the self-critical stance administrators generally took towards other aspects of the monitoring such as the overly broad and homogenizing classification “migration background” or the stigmatizing effects of the “deficits” highlighted by the indicators (see Petzke, 2021).

To be sure, as interviews with civil servants in Stadthausen revealed, they had grown increasingly skeptical of the indicator “fertility rate” – but only *after* voices from outside the administration questioned the indicator’s pertinence for measuring integration. As the head of the statistical office recalled: “We were asked, ‘is this a goal of integration that foreign women are giving birth to as many children as Germans, can that really be an indicator of integration,’ and we said, ‘well, when it comes down to it, no.’” The episode signifies how particular selections of indicators that were taken for granted within the executive committee did not make immediate sense to other actors and stakeholders.

In conclusion, the people responsible for the construction of integration monitoring instruments are heads of bureaucratic departments (for integration, social affairs, statistics, etc.) on the municipal, state, and federal level (*Amtsleiter* or *Referatsleiter*). In devising instruments for quantitatively monitoring integration, they often enlist the expertise of university professors, some of whom have established considerable renown as sociological consultants for integration policy. All of these actors generally belong to the same occupational category, so-called civil servants with executive duties (*Beamte im höheren Dienst*), a group that includes university professors, who, in Germany, are state-employed. Indeed, as higher civil servants, the responsible administrators within the bureaucracy themselves have an academic background, usually in one of the social sciences, in social work, or in law, and some even have a doctorate degree. As I argue, it is the habitus of this segment of social space that is implicitly universalized in measuring the extent to which lives are “integrated” in German society. At stake are cultural practices that, as I will show in the following, are prototypically “bourgeois” but are not reflected as class-cultural particularisms. On the contrary, civil servants are guided by an intuitive and self-evident “doxa” in selecting indicators, settling on a compilation which to them seems largely uncontroversial and which indeed is presented in this way in the publications. In fact, the reports explicitly disavow ethnocultural notions of assimilation and often stress the value of diversity and the preservation of one’s cultural identity. This makes them an especially pertinent case for analyzing processes of unwittingly transforming a particular cultural standpoint into a universal standard. In many ways, they represent a “least likely case” for cultural biases, given the heightened sensitivity to cultural impositions.

## Bourgeois culture in Germany

In order to gauge the class-cultural implications of some indicators in the integration monitoring, it is useful to employ as a backdrop what shall here be taken as an “ideal type” of bourgeois culture, following the work of Jürgen Kocka (1987, 1988, 1993) on the 19th-century German bourgeoisie.

Rather than sharply delineate a social group in socio-structural terms, Kocka (1987, p. 43) defines the *Bürgertum* of the 19th century through its “specific pattern of meaning and assessments, mentality and culture” (transl. by Volkov, 1993, p. 370), which integrated a heterogeneous mix of middle-class demographics. This culture includes: a “particular valorization of individual achievement,” which is tied to a “positive attitude towards regular work, a typical penchant for a rational and methodical life conduct”; the “pursuit of an autonomous arrangement of individual and mutual tasks, the latter in the form of voluntary associations”; an “emphasis on education (*Bildung*)” rather than religion; a “close affinity to aesthetic culture (art, literature, music)”; finally, a central understanding of family as a private, autonomous, and emotionally constituted unit with “few children and a sustained effort at their education” (Kocka, 1987, pp. 36, 43–44; my translations). This ideal-typical depiction of 19th-century bourgeois mentality and culture will be adopted here to assess the extent to which statistics in the integration monitors in fact implicitly subscribe to typically bourgeois values.

Crucially, Kocka has highlighted the significant ambivalence inherent in the German term “Bürger” that resonates with precisely the “double face of the state” as elaborated by Bourdieu (2014). “Bürger” connotes both the “citizen” as the universal subject of the state and the “bourgeois” as the member of a particular social stratum. In this sense, the project of *Verbürgerlichung* or “embourgeoisement” pursued by the *Bürgertum*, which saw itself as *allgemeiner Stand* or “general estate,” comprises a universalizing moment as much as a moment of distinction. The ambition to build an inclusive *bürgerliche Gesellschaft* as an order of free citizens that would do away with “absolutism, privileges of birth, and clerical manipulation” (Kocka, 1993, p. 9) was ambiguously intertwined with a project of assimilating other groups to the particular mores and habits of the bourgeoisie. The historical case of Jews in Germany makes this perfectly obvious:

“[It] demonstrates that the achievement of full civil rights (*Staatsbürgerrechte*) went hand in hand with the ascent into *Bürgertum*. Without the latter it was difficult to achieve the former. The demand for full equality as citizens was a demand that Jews could raise and gradually achieve only when they had become *bürgerlich* in their language and education, social manners and customs, their hygiene and their manner of dress” (Kocka, 1993, p. 10; see Volkov, 1993).

While the German bourgeoisie varied widely in its socioeconomic composition, at its core it was decisively shaped by its relationship to state officialdom, the importance and prestige of which “was striking even by Continental standards” (Blackbourn, 1991, p. 5). The growth of the German bourgeoisie was at its beginning closely linked to the growth of the state apparatus and the concomitant emergence of a class of educated officials, the *Beamtentum*. Indeed, the “Bildungsbürgertum,” or “educated bourgeoisie” was largely seen even by contemporaries as coextensive with academics in state-employment: bureaucrats, judges, university professors, Protestant ministers, and teachers (Bödeker, 1989, p. 25). In his *Philosophy of Right*, Hegel (1821) himself even identified the “general estate” or universal class with the class of civil servants. The free professions of independent doctors, lawyers, notaries, etc. arrived only much later on the scene, and even here the relationship with the state was a comparatively intimate one (Kocka, 1993, p. 30). This “bureaucratic mentality” (Kocka, 1993, p. 29) left a significant mark on the general outlook of the bourgeoisie even after the propertied classes expanded in the wake of the belated yet rapid onset of industrialization in Germany. Thus, in order to designate more precisely the central carriers of this cultural core, I use the term *Beamtenbürgertum* in this article. It forms a subsegment of the educated bourgeoisie (*Bildungsbürgertum*), with the latter comprising all occupations which, as opposed to the propertied bourgeoisie (*Besitz-* or *Wirtschaftsbürgertum*), derive their social position and life chances primarily from their academic credentials (Kocka, 1989, p. 9).

The assimilatory momentum of civil servants, especially, played a pivotal role in the inception of social statistics in Germany. The first social surveys arose in the context of the social question, later re-specified as the worker question, of the mid-19th century (Oberschall, 1965; Steinmetz, 1993). Not just in Germany but in most of Europe, the swelling working classes were regarded by the bourgeois establishment with a considerable fear of social upheaval. Crucially, while pioneers of social surveys in France and England predominantly hailed from the ranks of physicians or philanthropists, in Germany such initiatives “originated directly from the bureaucracy and the university professors who were mockingly known as the ‘socialists of the chair’” (Oberschall, 1965, p. 4). Indeed, some of the most seminal empirical research on the conditions of the workers was conducted by the *Verein für Sozialpolitik*, an association “dominated by academic economics, statisticians, and administrators” (Grimmer-Solem, 2003, p. 69) and thus by precisely the same segment of social space, higher civil servants, which is also behind today’s integration monitoring in Germany.

In an address delivered at the *Verein*’s inaugural meeting, Gustav Schmoller, a professor and historical economist and one of the *Verein*’s most influential figures, spoke quite candidly about the true concern behind the worker question. According to him, the problematic antagonism between the working classes and the propertied and educated classes was not so much an economic one; “more dangerous” was the “chasm in ethos, education, views, and ideals”; and here he pointed to the example of “civilizations as those of the Greeks, the Romans, and others” whose demise was brought about by “similar oppositions” (Schmoller, 1873, p. 4).

Indeed, in his treatise on the “Worker Question,” which he deemed the *Verein*’s de-facto political program (Grimmer-Solem, 2003, p. 138), Schmoller (1864, p. 525) made it clear that the elevation of the worker’s living standards was above all inhibited by his “ethical conditions,” invoking his tendency to live “only for the moment,” his little appreciation for a “comfortable home” and “adequate reading,” and his lack of a “sense of personal responsibility.”

Not surprisingly, then, early statistical initiatives in Germany such as those by the *Verein *guiding the “conciliation between the higher and lower classes” (Schmoller, 1873, p. 4) were deeply suffused with bourgeois cultural norms, dedicating a substantial part of their questionnaires to ethical behavior (Oberschall, 1965, pp. 19–21). Just as in the integration monitoring of contemporary Germany, the impetus behind such statistical observations lay in attenuating discrepancies between two population segments. Yet, in contrast with today’s efforts, this was consciously reflected as an endeavor of *cultural assimilation to the moral standards of the bourgeoisie*. This highlights how “going back to the genesis is very important, because there are debates at the beginning in which all kinds of things are said which later appear as the provocative revelations of sociologists” (Bourdieu, 2014, p. 63). It is here where the orthodoxy behind statistics on inequality and the integration of disadvantaged groups is still openly expressed and its contingency apparent before it is turned into a taken-for-granted doxa in “an amnesia of genesis” (Bourdieu, 2014, p. 184).

The extent to which bourgeois culture has been successful in its project of “embourgeoisement,” whether it persisted, eroded, or reemerged is a controversial matter (Conze, 2004; Kocka, 2008; Siegrist, 1994; Wehler 2001). Most would agree, however, that the bourgeoisie as a self-conscious formation has lost its contour as the antagonism with the aristocracy and the workers has faded. At the same time, many bourgeois values and practices are no longer exclusive to a circumscribed social stratum but widely shared, if to different degrees and in articulation with other class-cultural elements. The fully-fledged life-style of the educated bourgeoisie has thus ceded its universal pretensions and retreated into a sub-culture (Lepsius, 1992, p. 18). Despite its loss of influence, however, it perhaps still has its most fundamental anchoring in the apparatus of the civil service (Nolte & Hilpert, 2007, p. 46).

This last fact is crucial. Civil servants have a privileged position in defining universal standards, given that they control the means of exercising symbolic power. When “states state” (Corrigan & Sayer, 1992, p. 3; Bourdieu, 2014, p. 11), it is above all civil servants who are stating. In so doing, they “attempt to give unitary and unifying expression to what are in reality multifaceted and differential historical experiences of groups within society, denying their particularity” (Corrigan & Sayer, 1992, p. 3). As a result, such differences can only be thought of in “terms of the logic of deprivation” (Bourdieu, 1991, p. 53). Even without the orthodox militancy of a conscious agenda of embourgeoisement, and perhaps even more effectively so, civil servants are likely to shape official norms simply by acting on their habitual inclinations. As I show in the following, through their choice of indicators of immigrant integration, civil servants indeed enshrine a doxa of an integrated life in Germany to which *their* own lives correspond most closely to.

## The bourgeois undercurrent of monitoring integration

Given social statistics’ roots in an agenda of assimilation of the working class to the bourgeoisie, there are reasonable grounds to assume that such bourgeois legacies have potentially carried over into a statistical instrument that is likewise concerned with integration. There is, however, another compelling reason for such assumptions. As social statistics today continue to lie predominantly in the hands of civil servants, they are still liable to exhibit preconceptions of the *Beamtenbürgertum* as a particular segment of the educated bourgeoisie. As I argue, class-culturally particularistic preconceptions of the good life are indeed palpable in the compilation of indicators in the integration monitoring. Building on the historical ideal-type of bourgeois culture delineated above, I shall consider four ideal-typically bourgeois value-complexes that are tacitly universalized by such integration statistics: stability, self-reliance, and foresight; the privacy and self-sufficiency of the nuclear family; sociability and civic engagement; and politics and public discourse. I use data from the ALLBUS, the German General Social Survey, to illustrate the extent to which we are in fact dealing with an implicit universalization of civil servants’ ideas of the good life.^4^

### Stability, self-reliance, and foresight

As indicated above, bourgeois mentality is characterized by a particular regard for routine, continuity, steadiness, moderation, and predictability, the hallmark of Max Weber’s modern *Berufsmenschentum*. It thus comes as no surprise that a major component of the statistical instruments for measuring integration pertains to education and work (see Table 1). What is more, most of the monitoring reports on the municipal, state, and federal level provide justifications in the text for including a specific indicator. These often quite normative statements serve well to highlight the implicit ideals that inform such statistical perspectives on integration. Westlanden’s monitoring, for instance, pictures education and work as keys to an “independent life” and a “secure occupational position” that “guarantees a reliable income [and] makes life-planning possible.”

In emphasizing continuity, planning, regularity, and stability in this way, the attendant indicators in such sections do more than problematize the financially precarious and indigent circumstances of the disadvantaged. They at the same time implicitly devalue more alternative, non-committal, and “bohemian” lifestyles that are marked by experimentation, indulgence, and even contrarianism.

An indicator which compares the percentages of those owning (rather than renting) real estate among persons with and without a migration background further conveys this tacit regard for constancy and steadfastness (see Table 1). The mere selection inevitably suggests the relevance of this aspect of life, and the negative discrepancy between persons with and without a migration background wittingly or unwittingly indicates which of the two living arrangements is to be preferred. It is evocative of bourgeois norms of sedentariness, settlement, and commitment. Such valorizations implicitly hold a very particular and bourgeois life model to be the normative standard. In turn, lifestyles characterized by mobility, flexibility, and few attachments, whether they are purposefully chosen or not, are implicitly marked as aberrant.

Normative commitments to such ideas of sedentariness also came through in several of the civil servants’ comments and actions. For instance, the Stadthausener monitoring notes that the percentage among migrants owning real estate for personal use “is *not even* half” (my emphasis) of the proportion among native Germans. Promoting an increase in real-estate ownership is thus listed in the action plan of Stadthausen’s integration department. Another municipality in Westlanden includes the rate of fluctuation in its monitoring reports, which has even led to a political campaign advertising the city as a “location to stay” (interview with head of the integration department). Likewise, during a network meetings of integration officers in Westlandian municipalities, the mayor of the hosting municipality gave a welcoming address in which she noted that they wanted the people who migrate to their city “to put down their roots here” (fieldnotes).

However, in segmenting the population of German nationals in Germany into different occupational groups using data from the General Social Survey (ALLBUS), we find that such long-term commitments are particularly characteristic of the very people who construct integration monitorings.^5^ Given that those opting to live in a house generally tend to own it, owned apartments are a more suitable indicator for comparing sedentariness as such, independent of types of real estate. And indeed, when looking at all German nationals who live in apartments, civil servants with executive duties have the highest share of those owning the apartments they live in compared to all other occupational groups, at 24% (see Table 2).

There is a specific phenomenological relationship to time at stake with many of these indicators. It is the typically bourgeois penchant for long-term planning, the methodical and rational pursuit of distant ends, deferred gratification, and an investment into the future.^6^ Such time-perspectives are to a significant degree “classed” (Bourdieu, 1984, pp. 296–297, 2000, pp. 206–245).^7^ It is first and foremost the ascending fractions of the middle class that exhibit an ascetic, far-sighted, and rational perspective on time since it is they who have the most to gain from it.

Even such innocuous and uncontroversial indicators as the use of preventive health measures among persons with and without a migration background implicitly cast such temporal perspectives as the normative standard (see Table 1 for examples). Again, data from the ALLBUS indicates that this is associated with class. Table 2 shows the percentages of people within the different occupations who have visited a doctor for preventive care or vaccinations within the last 3 months. Civil servants with executive duties have the highest proportion of those within an occupation who have visited a physician for such reasons.^8^

Civil servants also exhibited such valorizations of foresight and future-planning in interviews. The head of Stadthausen’s department of economic affairs, who is also a member of the executive committee responsible for the monitoring, was palpably bewildered that immigrants showed comparatively little inclination to avail themselves of the free consultations his department offers to business start-ups:

The typical founder who is being advised by our agency... comes here and makes a business-plan and goes on for I don’t know how long in deliberating [legal titles such as] ‘LLC’ or ‘registered merchant’…and does every conceivable thing first. Some of the ethnic groups function differently…Here one doesn’t go to the classic consultancy agency for business start-ups. One simply goes ahead and opens the business.

The comment also exemplifies well how such differences are generally attributed to ethnicity or migration background rather than to class.

### Privacy and self-sufficiency of the nuclear family

Several indicators in the integration monitoring systems on the municipal, state, and federal level present data on the size and composition of households and/or on fertility rates (see Table 1). As they do so, they highlight the relevance of the family and inadvertently valorize a specific model of family life over others. Inevitably, in the context of an integration monitoring focused on integration deficits, the generally larger family structures of migrants are implicitly problematized in their juxtaposition to the more “nuclear” structures of non-migrants. This resonates with bourgeois ideals. In fact, the 19th-century *Bildungsbürgertum* “invented” the idea of the private family circle secluded from public, i.e., economic or political, affairs (Kocka, 1987, pp. 36, 43). Comparisons of household size evoke its distinction from “older forms of communality in the extended family which continued to be observed among the ‘people’…until long after the eighteenth century” (Habermas, 1991, p. 44).

Moreover, the focus on the number of children further validates the calculative time-perspectives typical of the educated bourgeoisie. Again, it is precisely in this class segment where birth control originated (Nipperdey, 1990, p. 384). Less children allow for a more concentrated transmission of culture and for more resources to be channeled into their social attainment, something that is obviously of less relevance to the already established upper classes and less feasible, because comparatively less likely, for those on the lower rungs of society.

Indeed, the rationale for including this indicator in the monitoring, given by the head of Stadthausen’s department of integration, is in many ways telling: “For us the indicator was important to see – If you have families with many children, you can presume that the financial situation is more difficult than when one has less children, that the living space needed is a different one and so on. So, it was a planning approach.” Ironically, in selecting this indicator with an eye toward “planning” integration policy, the civil servants were considering precisely those issues which, as noted, members of the educated bourgeoisie would typically take into account in their own family “planning.”

However, data from the ALLBUS again substantiates that the extent to which such perspectives are shared varies widely among German nationals themselves. Among the German population above the age of 50, when, presumably, families have largely reached or come close to their final size, civil servants with executive functions have a number of children almost identical to the total mean. In that, they clearly stand apart from farmers, unskilled and low-skilled workers, as well as from the major entrepreneurs of the upper class (see Table2). The equivalence with the general mean indicates that civil servants value family in that they generally do have children (as is also indicated by the inclusion of this indicator) but not as many children as those on the top and bottom rungs of economic prosperity.^9^

**Table 2:** “Integration indicators” by occupational group

Occupational Group	Living in self-owned apartment (%) ^a^	Prophylactic med. exam in last quarter (%) ^b^	Avg. children at age 50+ (biol., step, adopt.) ^c^	Civic engagement once a week or more (%) ^d^	Politics and public life very important (%) ^e^	Interest in politics very high (%) ^f^	Political engagement very important (%) ^g^
Independent farmers	19.1	27.1	2.53	16.0	24.6	6.9	22.2
Independent professionals:		31.6					
with one employee or less	18.9	-	1.58	30.0	39.3	22.2	11.4
more than one employee	14.5	-	1.78	25.0	33.0	25.7	19.4
Self-employed in trade or craft, industry, or service:							
with one employee or less	13.4	29.3	1.70	14.4	25.0	12.3	17.2
with two to nine employees	20.5	30.5	1.80	19.9	24.9	12.7	15.9
with more than nine employees	22.8	13.3	1.98	20.2	36.0	19.1	26.3
Civil servants:							
carrying out simple/mid-level administ. duties	11.0	28.1	1.65	19.5	29.8	10.5	18.9
carrying out senior administrative duties	19.4	33.3	1.74	31.7	33.0	22.7	17.1
** carrying out executive duties**	**24.0**	**36.8**	**1.81**	**29.7**	**41.9**	**33.1**	**27.8**
Master craft- and tradesmen and foremen	13.2	23.0	1.83	21.4	28.0	10.3	14.2
Employees:							
with simple duties	8.9	27.2	1.86	10.0	21.5	4.1	5.2
under loose supervision carrying out complex tasks independently	12.4	30.8	1.68	16.9	25.6	8.1	9.6
carrying out responsible tasks independently	15.2	31.0	1.73	21.3	29.6	14.7	14.1
w./ manag. respons. & decision-making powers	16.6	36.7	1.78	22.3	38.5	21.5	19.4
Workers:							
unskilled worker	5.5	15.3	2.31	7.9	18.1	3.0	10.4
semi-skilled worker	6.9	17.2	2.05	9.6	20.7	4.0	7.9
skilled worker	7.2	19.9	1.81	12.7	22.2	6.5	11.9
Total	11.2	27.1	1.82	16.4	25.4	9.8	12.5

NOTE. – Base: German nationals except where otherwise indicated; weighted data.

Percentages for each item are based on totals within each occupational group.

^a^ N = 25,477 (27,910 unweighted) / ALLBUS 1980–2014 / Base: German nats. living in apts.

^b^ N = 3922 (3975 unweighted) / ALLBUS 2004, 2014 / Indep. prof. grouped as one so that N>=30

^c^ N = 11,019 (11,266 unweighted) / ALLBUS 2000–2014 (biannual) / Base: German nats. aged 50+.

^d^N = 11,159 (11,287 unweighted)/ ALLBUS 1998, 2002, 2004, 2012

^e^ N = 22,234 (22,377 unweighted) ALLBUS 1980, 1982, 1986, 1990, 1991, 1992, 1998

^f^ N = 51,327 (51,790 unweighted) ALLBUS 1980–2014 (exc. 1988)

^g^ N = 5727 (5787 unweighted) ALLBUS 2002, 2012

### Sociability and civic engagement

Most of the integration monitorings devote much space to the question of how civic engagement among persons with a migration background compares to persons without a migration background. Data for involvement in voluntary associations, political parties, and sports clubs come mostly from national survey research and the Socioeconomic Panel (SOEP). In the context of discussing the process of selecting indicators, we have already seen that civil servants unreservedly point to a “civic sense of responsibility” and an expectation of “contributing to society” in justifying such a focus.

However, in surveying civic engagement, especially in such associational forms, a very particular and bourgeois conception of the good life is once again implicitly presented as universal. The German term for voluntary associations, *bürgerliche Vereine*, once again mirrors this ambivalence. In themselves, they are an embodiment of core values of the 19th-century bourgeoisie (Eisenberg, 1993, p. 154; Nipperdey 1976, p. 181). Originally, they were seen as means to foster sociability for the “purpose of ‘serious,’ ‘rational,’ and ‘useful’ discourse, for united edification…or joint activities for the common good” (Ruppert, 1984, p. 131). As such, they were at the same time regarded as a way to mix and culturally assimilate the classes by “transmitting bourgeois values to the sub-bourgeois strata” (Eisenberg, 1993, p. 154).

Consequently, one would expect associational life in Germany to be still most centrally situated in the bourgeois milieu. Survey research in Germany indeed identifies civil servants as the occupational category with the highest share of those pursuing volunteer work (Schwarz, 1996, p. 264). Data from the ALLBUS can locate such engagement more specifically in the upper echelons of the civil service. As Table2 shows, along with the free professions with up to one employee, the two most senior categories of civil servants once more stand apart as the occupational group with the highest proportion of those engaging in such work at least once a week or even daily.

### Politics and public discourse

The implicit normalization of civic engagement and associational membership resonates with a bourgeois ideal of rational discourse and deliberation for the sake of progress and the autonomous governance of society by free and equal citizens (Eisenberg, 1993; Nipperdey, 1976). Unsurprisingly, then, the degree of political activism is also measured in the monitoring reports by indicators on the frequency of participating in political parties, local politics, or citizens’ action committees/grassroots initiatives (see Table1).

Research has, of course, long pointed to a marked class-based variance in political engagement, with more highly educated people in higher income brackets typically exhibiting more political engagement both in the form of voter participation as well as more sophisticated political activities such as participation in political initiatives, parties, and organizations (Brady et al., 1995; Verba et al., 1978). Culturalist explanations of such variances have cited notions of “civic duty” (Campbell et al., 1960) or a felt “obligation to participate” (Almond & Verba, 1963, pp. 161–179), which, as seen, were invoked almost verbatim by the civil servants themselves. These factors may indeed be understood as particularly bourgeois. They not only carry an air of “noblesse oblige” regarding civic engagement but are also indicative of deeply ingrained beliefs of “political efficacy” (see Abramson, 1983, pp. 135–189 for an overview), i.e., of being at the helm of the social process and seeing oneself as a part of a common social project.

A closer investigation using data from the ALLBUS can locate the stronghold of political engagement within social space in an even more exact fashion. Table2 shows percentages of those within an occupational group assigning high importance to the sphere of politics and public life, professing a high interest in politics, and assigning high importance to political engagement. Among all occupations, civil servants with executive duties again show the highest shares within their occupational group on each of the three indicators.

Just as in the 19th century, then, when civil servants first launched surveys with the aim of integrating a disadvantaged group, today’s indicators are deeply imbued with a conception of the good life most strongly held by the *Beamtenbürgertum* as a core segment of the educated bourgeoisie. What has changed is the degree to which these cultural particularisms are reflected. Indeed, the bourgeois “orthodoxy” explicit in early monitorings of workers’ lives has become settled “doxa” in today’s instruments of measuring immigrant integration.

## Concluding discussion

This article adds to a recently reinvigorated literature that mobilizes perspectives from cultural sociology to explore social inequality and immigrant incorporation. While such work has broken new ground conceptually and empirically, rarely has it turned its view to the culture of those constructing the problem of socioeconomic mobility itself.

This article has addressed this neglect by mobilizing Pierre Bourdieu’s cultural sociology of the state. This framework advances extant literature in several ways. For one, it allows for a *relational* perspective that situates the construction of social inequality in an ecology of class positions. Second, by underscoring the state’s monopoly on legitimate symbolic violence, it illuminates how the very terms of the debate on social inequality and immigrant integration are set by and filtered through conceptual schemes rooted in the habitus of civil servants with executive functions. Third, in employing the concept of symbolic domination, it does more than merely highlight biased valorizations of certain class cultures. It argues that in constructing not just orthodoxy but doxa, the perspectives of the state essentially constitute an *erasure* of alternative forms of life conduct. Dominated lifestyles now necessarily fall along the line of a one-dimensional scale of proximity to the unquestioned standard established by the state. In consequence, cultural alternatives can “only be conceived in terms of the logic of deprivation” (Bourdieu, 1991, p. 53).

In focusing on Germany’s integration monitoring, this article has specifically attended to the complicity of statistical expertise in the state’s construction of disadvantaged lives, enlisting perspectives of the history and sociology of quantification. The monitoring reports unwittingly impose a normative view on what ought to be considered a standard biography in Germany. They do so not just by measuring deviations from statistical means but more fundamentally by their particular choice of indicators. Measuring a specific dimension already grants a marked significance to the dimension *per se*. The configuration of indicators thus establishes a selective statistical grid in which individual existences are placed. To the extent that such grids are informed by taken-for-granted notions of worthy and reputable lives, they end up measuring little more than distances from a particularistic ideal. In so doing, they obscure, even entirely eclipse, different models of the good life which arguably would entail entirely different selections of statistical indicators. The selectivity of the indicators is in fact thoroughly concealed by the scientific objectivity they convey.

The normative model conveyed by these statistics is much in line with an ideal-typical bourgeois subject culture as it emerged in the 19th century. It favors lives marked by financial independence, methodical life-planning and foresight, security, sedentariness, long-term commitment, intimate and nuclear family structures, civic engagement and sociability, as well as political responsibility and public deliberation. Following Bourdieu, however, it can be seen as situated in a specific position in social space and as merely one position-taking in a space of possibles of heterogeneous lifestyles, tastes, and orientations.

The fact that administrative officials and the social-scientific expertise they employ unwittingly construct integration deficits as deviations from a class-culturally particularistic view of the good life is not without irony. Official definitions of integration espoused in the statistical reports define integration purely as an “equalization of life situations” and often distance themselves from implications of (ethno-)cultural assimilation in stressing the “preservation of one’s cultural identity.” Yet, statistical indicators of the integration monitors impose a starkly uniform “culture” of civil servants. What is implicitly affirmed under “diversity,” then, is the paradoxical notion of a “bourgeois diversity.” What is tacitly imagined, it seems, are people utterly uniform in their values and aspirations. Integration statistics essentially portray a view of immigrants as “inconvenienced civil servants.” This example thus calls for closer attention to the ways in which stylizations of the problem of “disadvantaged” lives are steeped in a particular class-culture and overdetermined by relations of observer and observed in social space.

In fact, given the state’s monopoly on symbolic violence, such stylizations may obfuscate the actual extent to which such bourgeois conceptions of the good life have lost traction even among the broader middle-class. There are indications that the particular ideal conveyed by integration statistics may hark back to a society that no longer exists and to subjectivities that are increasingly on the wane. As argued above, the monitorings implicitly subscribe to a model of life characterized by stability, long-term planning and commitment, risk-aversion, and sedentariness. In contrast, sociologists have argued that today’s economy fosters and even demands subjectivities that are first and foremost marked by flexibility: i.e., a disposition towards short-term rather than long-term commitment; high tolerance – even affirmation – of risk, change, and mobility; and more fleeting, superficial ties to people and places (see, e.g., Sennett, 1998). Boltanski & Chiapello (2005) even describe the rise of a “new spirit of capitalism,” resulting from capitalism’s ingestion of an “artistic criticism” that was in no small part a critique of bourgeois morals. In this order of worth, stability, rootedness, and a preference for security in fact indicate moral inferiority (ibid., pp. 119 − 21).

Immigration itself is surely related to these developments. Highly qualified migrants, especially, are likely to correspond closely to such subjectivities, making them highly mobile and perhaps only temporary residents of their host-country. Indeed, some of the tectonic forces that drive migration – greater economic and political interdependencies, technological innovations in transport and communication – are certainly also crucially involved in the emergence of new subjectivities that make a virtue out of the economic repercussions of such changes.

It is for this reason that the perspectives of the integration monitoring may be to a large extent quixotic, even paradoxical. They implicitly aim to assimilate migrants to an ideal subjectivity which may actually be eroding due to similar circumstances that fuel at least some forms of migration. And indeed, studies have pointed to a continuous “dissolution of the middle” in Germany, be it in economic terms (e.g., Grabka & Frick, 2008; Mau, 2012), or, more importantly, in cultural terms of a displacement by more uncommitted, hedonistic, and non-bourgeois segments (Hradil & Schmidt, 2007, pp. 212–15, 219–20; Vester et al., 2001, pp. 328–369). Ironically then, the means to create and impose standard measures of integrated lives lie in the hands of a group whose own ways and orientations are in fact becoming less and less exemplary.

## Footnotes

1 On the relation of Bourdieu’s theory of the state and Gramsci’s notion of hegemony, see Burawoy (2019).

2 Saeed (2021) similarly argues that the ways in which symbolic power is exercised by the state has been undertheorized. See also Loveman (2005), who tries to shed more light on the ways in which the state accumulates symbolic power in the first place. For a critique regarding an unresolved tension between Bourdieu’s conception of the state as a field and the state as unmoved mover guaranteeing a symbolic order, see Schinkel (2015) and Loyal & Quilley (2017).

3 See also Elrick & Winter (2018), who present an interesting and in many ways complementary study on how various immigration policies in Germany, from visa waivers to family reunification, inadvertently privilege immigrants from the middle class.

4 To be sure, notions of the good life can only be directly inferred from those indicators that survey a particular ethical stance (such as the subjective importance of political engagement). For other indicators, such notions can only be indirectly inferred from self-reported behavior or living conditions that mirror personal choices. However, while it is important to distinguish between attitudes and actual conduct, it seems less reasonable to assume that such behaviors run counter to personal ideas of the good life than to assume that they align. See Vaisey (2010) for a recent contribution on attitudes and behavior that supports this line of reasoning.

5 For the occupational differentiation, a slightly condensed version of the variable “Berufliche Stellung: Einordnungsberuf nach Terwey” [occupational meta-classification according to Terwey] was used (v864). See GESIS (2016, pp. 1478–1483) for details.

6 Cf. Lyman & Scott (1989, pp. 45–46) on the perception of “hippies” as a threat to the social order which is precisely due to their withdrawal from hegemonic regimes of time; see also Thompson (1967/2018, p. 36).

7 The classic reference for time orientation is LeShan (1952) and for deferred gratification Schneider & Lysgaard (1953).

8 The independent professions are classified as one to ensure that N > = 30 for each occupational group. Types of medical insurance, for which data was not available, may play a role here.

9 When not filtering for age, civil servants with executive duties are above the mean (1.61 with a mean of 1.45).
